# A Multiphase Semistatic Training Method for Swarm Confrontation Using Multiagent Deep Reinforcement Learning

**DOI:** 10.1155/2023/2955442

**Published:** 2023-07-07

**Authors:** He Cai, Yaoguo Luo, Huanli Gao, Jiale Chi, Shuozhe Wang

**Affiliations:** School of Automation Science and Engineering, South China University of Technology, Guangzhou 510641, China

## Abstract

In this paper, we propose a multiphase semistatic training method for swarm confrontation using multi-agent deep reinforcement learning. In particular, we build a swarm confrontation game, the 3V3 tank fight, based on the Unity platform and train the agents by a MDRL algorithm called MA-POCA, coming with the ML-Agent toolkit. By multiphase learning, we split the traditional single training phase into multiple consecutive training phases, where the performance level of the strong team for each phase increases in an incremental way. On the other hand, by semistatic learning, the strong team in all phases will stop learning when fighting against the weak team, which reduces the possibility that the weak team keeps being defeated and learns nothing at all. Comprehensive experiments prove that, in contrast to the traditional single-phase training method, the multiphase semistatic training method proposed in this paper can significantly increase the training efficiency, shedding lights on how the weak could learn from the strong with less time and computational cost.

## 1. Introduction

**Figure 1:**
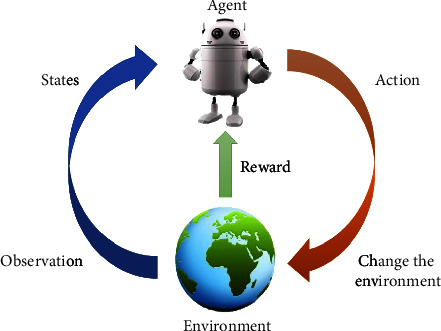
How agents learn by reinforcement learning.

Inspired by behavioral psychology, reinforcement learning (RL) is an important machine learning method by mimicking the learning patterns of humans and animals from the perspective of reward and punishment [1]. As shown in Figure 1, in reinforcement learning, the agent perceives information about the surrounding environment and takes actions it deems appropriate, which will in turn bring back rewards or punishments from the environment that will affect the agent's future decisions [2]. The ultimate goal of the agent is to find the policy that maximizes the reward value [3].

The concepts and terms of reinforcement and reinforcement learning were first proposed by Minsky [4] in 1954. Years later, Waltz and Fu brought this concept to the control community [5] and made it clear that the core mechanism of reinforcement learning is trial and error by means of reward and punishment. In 1957, Bellman solved the Markov decision process with the idea of reinforcement learning [6], making the Markov decision process the most common form of defining reinforcement learning problems. Q-learning proposed by Watkins further expanded the application of reinforcement learning, which has become the most widely used reinforcement learning method today [7]. However, the development of reinforcement learning technology became slow in the 90s, obscured by the light of supervised learning. The guidance with external supervision and preparatory knowledge of supervised learning is completely different with the philosophy of reinforcement learning. Reinforcement learning has thrived again since 2013 marked by the historical DeepMind's paper which adopted reinforcement learning to play Atari games. From then on, with the rapid development of high-performance computing, big data, and deep learning technologies, deep reinforcement learning (DRL), which integrates deep learning with reinforcement learning, began to emerge, especially in the field of games. The agents trained by Mnih et al. [8] are able to successfully learn from visual perception consisting of thousands of pixels, and the agents obtained by this method performed at a level comparable to that of experienced players in Atari 2600 games. As is known to all, the AlphaGo robot developed by Deepmind defeated the world Go champion Lee Sedol [9]. Furthermore, the AlphaGo Zero, based on deep reinforcement learning, easily defeated AlphaGo after a short period of training with complete self-learning [10]. In 2017, the agent created by Brown and Sandholm defeated the world's top players at the game of Poker [11]. Besides the field of games, deep reinforcement learning has also made remarkable achievements in other areas such as autonomous driving [12] and robot control [13].

The great success of deep reinforcement learning has encouraged researchers to explore more complex multiagent systems (MAS). Multiagent system refers to a distributed intelligent system composed of multiple agents which is expected to achieve some global goals [14]. By taking the MAS as system models, the structure, function and behavior characteristics of the system are expressed through the communication, coordination, distributed scheduling, management and cooperative control among the agents. MAS has many advantages, such as autonomy, distribution, coordination, self-organization, cooperative learning, and reasoning [15], which makes it a promising solution to practical application problems with high robustness, reliability, and efficiency. A MAS endowed with reinforcement learning algorithms can independently explore and find the optimal solution for the team through the interactions between agents as well as the interactions between agents and the environment. In this way, no human intervention is required to program an agent with complex strategies to accomplish the specific goals. The ability of MAS to seek optimal solutions in the face of complex environments is unmatched by other methods, which can provide a unified model for various practical situations for most cases. In order to solve problems under more complex multi-agent environments, researchers applied DRL to the MAS, which gave birth to Multiagent Deep Reinforcement Learning (MDRL). In the early stage, Tampuu applied the famous DRL algorithm, i.e., the DQN algorithm to the Atrai game [16] and successfully realized cooperation and competition between agents by adopting self-play learning [17] and two different reward strategies. However, this method does not consider the communication between agents and each agent regards other agents as part of the environment, thus leading to the problem of a nonstationary environment which makes the learning efficiency rather low. In recent years, with more efforts devoted to the MAS environment, several novel MDRL algorithms have been proposed. In [18], Sukhbaatar and Fergus proposed a MARL algorithm called CommNet, which realizes real-time communication between agents and shows better performance in contrast to the algorithms without communication. In [19], Lowe proposed the MADDPG algorithm, which is a policy gradient algorithm based on the actor-critic framework and has effectively solved the nonstationary environment problem. In [20], Sunehag et al. proposed the VDN algorithm, which adopted the idea of value function decomposition and successfully solved the problem of multi-agent credit assignment [21]. Later, Son proposed an improved VDN algorithm named QTRAN [22], which used a nonlinear matrix decomposition method for the *Q* function and further improved the learning performance. Nowadays, through the most advanced MARL algorithm, agents has exhibited a high degree of swarm intelligence and can even beat human masters. In 2019, MDRL based OpenAI Five, launched by the OpenAI team, defeated the world champion OG team in the 5v5 mode of the DOTA2 game [23]. Though human players could still beat OpenAI Five under certain constraints, the triumph of OpenAI Five proves to the world the great prospects of MDRL.

In this paper, we propose a multiphase semistatic training method for swarm confrontation using MDRL. In particular, we build a swarm confrontation game, the 3V3 tank fight, based on the Unity platform and train the agents by an MDRL algorithm called MA-POCA, coming with the ML-Agent toolkit. In the training process, for the scenario where a weak team is fighting against a strong team, the traditional single-phase training method for the weak team does not perform well. Specifically, the weak team sometimes learns nothing from the strong team and keeps being defeated, and for the cases that the weak team managed learning from the strong team, the train process would be extreme time-consuming with low training efficiency. Motivated by this observation, in this paper, a novel training method is proposed featuring multi-phase learning and semistatic learning. By multiphase learning, we split the traditional single training phase into multiple consecutive training phases, where the performance level of the strong team for each phase increases in an incremental way. On the other hand, by semistatic learning, the strong team in all phases will stop learning when fighting against the weak team, which reduces the possibility that the weak team keeps being defeated and learns nothing at all. Comprehensive experiments prove that, in contrast to the traditional single-phase training method, the multiphase semistatic training method proposed in this paper can significantly increase the training efficiency, shedding lights on how the weak could learn from the strong with less time and computational cost.

## 2. Preliminary

The ML-Agent Toolkit is a reinforcement learning toolkit developed by Unity Technology. Based on the Unity platform, ML-Agent Toolkit enables the game scene to be used as a training environment for reinforcement learning, which considerably facilitates the work of researchers.

The most important component in ML-Agent Toolkit is the learning environment, which consists of two parts: the agent and the behavior.

- Agent: the agent can be any object in the game scene. They collect observations from the environment at all times and take actions accordingly. They may get a positive or negative scalar reward value after taking actions. In addition, each agent is endowed with behaviors.
- Behavior: behaviors are the characteristics which describe the agent. Each behavior is marked by a unique Behavior Name. The role of the behavior is to return the desired action based on the observation and reward of the agent. Behavior include three types: Learning, Heuristics, or Inference. By default, behavior is assumed to be of the type of Learning.

In addition, the ML-Agent Toolkit also includes some other important components, such as the python low-level API, external communicator, python trainers, and Gym wrapper [24].

For now, the main reinforcement learning algorithms supported by ML-Agent Toolkit are SAC, PPO, and MA-POCA algorithm. Besides, the ML-Agent toolkit also supports online/offline learning and behavioral cloning techniques. These algorithms and techniques can be applied to symmetric and asymmetric self-play. SAC and PPO algorithms support options Module and Long-Short-Term Cell to extend algorithms and strategies by Intrinsic Curiosity [25].

MA-POCA is the latest reinforcement learning algorithm released by Unity Technology, which is devoted to multiagent reinforcement learning. This algorithm learns a centralized value function to estimate the expected discounted return of the group and a centralized agent-centric counterfactual baseline to achieve credit assignment in the manner of COMA [26]. Interestingly, MA-POCA algorithm does not directly train the agent, but trains an “instructor,” which is a centralized critic. The instructor teaches the whole team, rather than any individual [27]. Rewards can be given to the entire team, and the team will help the individuals to reach their goals. Furthermore, MA-POCA uses self-attention over active agents in the critic network [28], thus solving the problem of posthumous credit assignment without the need for absorption states [29]. Through the MA-POCA algorithm, even if the agents died in the confrontation, they can still know whether their actions are beneficial to the team's victory, and in this way they can learn and take actions that are more beneficial to the team in the next confrontation. Sometimes they even resort to self-sacrifice to help the team win.

## 3. Experiment and Results Analysis

**Figure 2:**
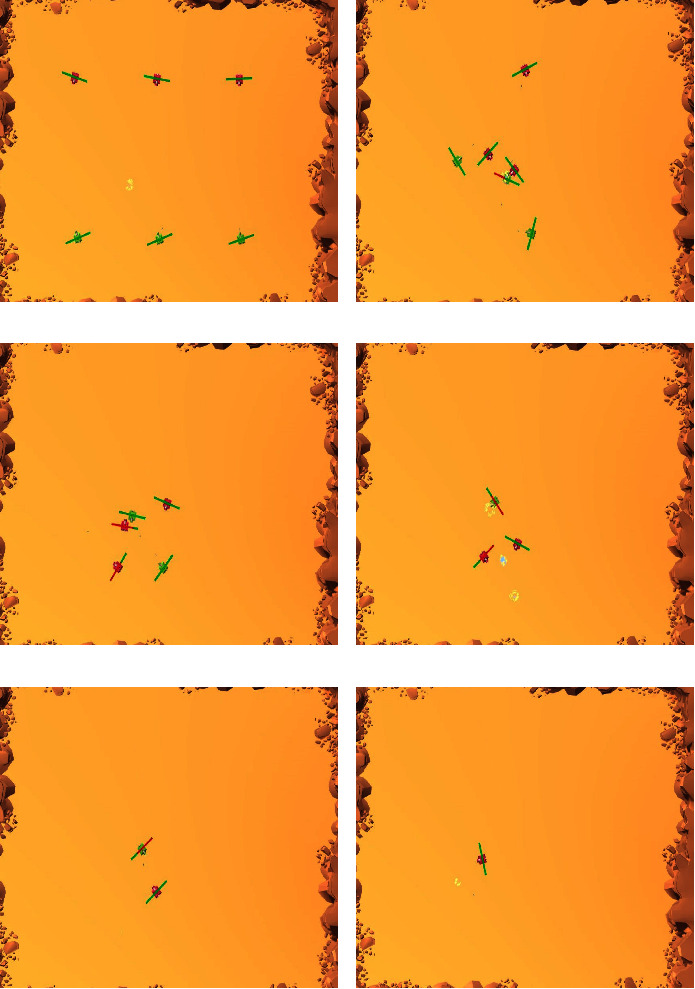
A typical complete game process. (a) Game kicks off. (b) Tanks start to confront. (c) A green tank is destroyed first. (d) Both teams lose one tank at the same time. (e) Another red tank is destroyed. (f) The red team finally wins.

In this section, we will introduce the environment setting, conduct experiments and analyze the associated results. The basic idea is to compare the learning performance of the single-phase training method and multiphase training methods subject to different “turning steps.” The team to train is a model finishing 10 M (For ease of expression. The authors will abbreviate “million” as “*M*” in the following text; for example, 100 millions is expressed as 100 M.) steps of self-training. In the training process, the strong team stops learning, and that explains why the training method is called semistatic. Winning rate is adopted as the main index to examine the learning performance.  Figure 2 shows a typical complete game process.

### 3.1. Environment Setting

In this paper, the simulation environment is built based on the Unity platform. Unity not only has a powerful graphics rendering function but also has the characteristics of fast distributed simulation features, which can significantly improve the learning efficiency.

**Figure 3:**
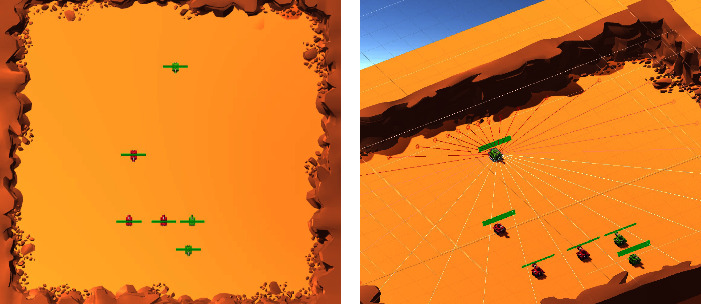
Environment setting. (a) The experiment scenario. (b) Ray perception.

The experiment scenario is designed as a flat rectangular area. In order to prevent the tanks from driving out of the area, walls are set around the area. Tanks are divided into red and green teams, each of which has three members. The positions of tanks are random when games begin, as shown in Figure 3.

In our experiment scenario, tanks can only move on the *X*-*Z* plane. The goal for the tanks is to eliminate as many enemy tanks as possible and retain their vitality by moving and firing shells. The rules of the game are described as Algorithm 1.

It is necessary to set reasonable attributes for tanks. The basic attributes include health, speed, shell damage, and vision. The attributes of a tank are summarized in Table 1, and all the tanks have the same attributes. It should be noted here that the actual initial velocity of the shell is the superposition of the tank speed and the firing speed, so the maximum can reach 30 m/s without failing to catch up with the enemy.

In addition, we adopt a sensor called ray perception sensor to help the tank perceive other tanks and walls. When a tank is equipped with this sensor, a number of rays will be generated starting from the origin of the coordinate system of the tank, and these rays will be emitted outward at a uniform angle, which can occluded by entities. As shown by Figure 3(b), when these rays hit an entity, the tank can get the entity's tag, say, teammate, enemy or wall. The relevant parameters of the ray perception sensor are given by: rays per direction: 15; sphere cast radius: 0.5 m; ray length: 70 m; stacked raycasts: 5; start vertical offset: 1 m; end vertical offset: 1 m.

### 3.2. Experiment Models

In this paper, semistatic training means in the training process between a strong team and a weak team, the weak team will keep learning, while the strong team will not. Now, we introduce the following models.

- Model Z: trained for 100 M steps in self-play mode in the first stage. In the second stage, increase the *Maximum* *HP* of the current model from 15 to 20. Let this strengthened model be the strong team, and the original model be the weak team. Train the weak team against the strong team in the semi-static way for another 80 M steps;
- Model 0: trained for 10 M steps in self-play mode, not converge;
- Model 1: on the basis of model 0, trained for 96 M steps in self-play mode;
- Model 2: select model 1 as the weak team and model *Z* as the strong team for semi-static training. We get model 2 after 24 M steps.
- Model 3: select model 1 as the weak team and model *Z* as the strong team for semi-static training. We get model 3 after 119 M steps.

All these models are trained by the MA-POCA algorithm as aforementioned. In the self-play mode, the tanks will fight against themselves and update the model at regular intervals. Model 0 is trained only for 10 M steps, and at this stage the reward curve for Model 0 has not yet converged as Model *Z* does. In other words, Model 0 is a beginner with some basic knowledge about the game, which makes it a suitable model to be further trained by the method proposed in this paper.

**Figure 4:**
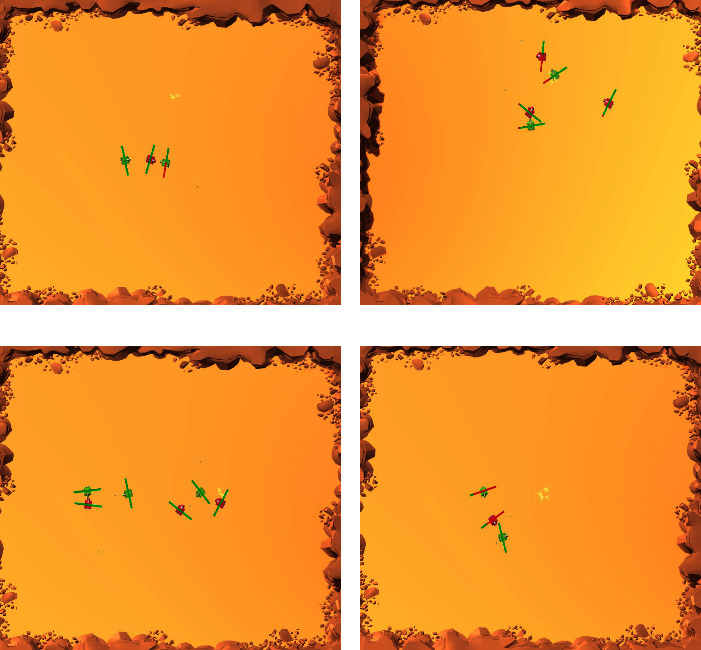
Intelligent behaviors of tanks in the confrontation. (a) Front and rear double-team (b) Go around behind the enemy and attack. (c) Besiege the minority by the majority (d) Luring the enemy to create attack opportunities for teammates.

In the following experiments, we choose Model 0 as the week team, and choose Model 1, 2, and 3 as the strong team. The initial confrontation information of the three strong teams vs. the weak team is given in Table 2. The criteria for the selection of the weak team is as follows. On one hand, the weak team cannot be too strong since it will deviate from the concern of this paper which studies how to learn quickly between two models with huge gap. On the other hand, if the pretraining is overinsufficient, the model will just be too weak to learn anything from any other model. In the training process, the models can sometime exhibit intelligent behaviors. For example, tanks will enclose the enemy to achieve favorable position. They also know that it is better to attack from behind. In some cases, one tank will lure the enemy, while other teammates attack in a sneaky way. Some typical intelligent scenes are shown in Figure 4.

### 3.3. Reward Strategies

For multiagent reinforcement learning algorithms MA-POCA, the behaviors of each agent are mutually influenced. We need to set rewards not only for a single agent, but also for the whole team. Without any doubt, reward setting greatly affects the training performance, including both the design of the reward items and their associated values. In our case, since group winning is the ultimate goal for the game, it is endowed with a relatively large reward in comparison with the rewards gained by intermediate and individual behaviors of the agents. Moreover, in addition to the final result of winning or losing, the group damage and battling time are also taken into consideration for reward calculation. Specifically, small group damage and short battling time will bring extra rewards to the winning team. It is worth mentioning that the absolute reward values are immaterial in training, but the relative reward values assigned for different individual and group behaviors matter. In the official example, the reward value is usually set less than 1. Tables 3 and 4 present our main reward strategies, where the terminologies *HP*, *FullHP*, *ResetTimer*, *MaxEnvironmentSteps*, and *GroupHP* denote the current health of each tank, the maximum health of each tank, the current game steps, the maximum game steps, and the sum of *HP* of all teammates, respectively.

### 3.4. Parameters Configuration

All reinforcement learning training in the experiment is configured using the same configuration file to make the obtained data convincible. Since the experimental scene in this paper is much more complex than the case for a single agent, it is important to set suitable training parameters such as higher *Num* *layers* and smaller *Time* *horizon*, where the specific parameters configuration are shown in Table 5. The training parameters of Table 5 are explained as follows.

#### 3.4.1. Hyperparameters

  Batch size refers to the amount of data required in each gradient descent iteration, which should always be multiple times smaller than *Buffer* *size*. In general, the more complex the actions are, the larger the batch size should be. In reinforcement learning, batch size should be large (on the order of 1000 s) for continuous actions, and small (on the order of 10 s) for discrete actions.
  Buffer size (default = 10240) refers to the amount of data required before each policy model update, which should always be multiple times smaller than *Batch* *size*. Typically, large buffer size corresponds to more stable training updates, yet slower training speed.
  Learning rate (default = 3*e* − 4) represents the initial learning rate for gradient descent, which corresponds to the strength of each gradient descent update step. Learning rate should be reduced if the training is unstable.
  Beta (default = 5.0*e* − 3) represents the strength of the entropy regularization, which makes the policy more random, ensuring that agents properly explore the action space during training. Increasing beta will result in more random actions, but too many random actions may not lead to a good result. Beta should be adjusted such that the entropy (measurable from TensorBoard) slowly decreases along the increase in reward. If entropy drops too fast, beta should be increased, and vice versa.
  Epsilon (default = 0.2) is a factor that affects how fast a policy develops during training, which corresponds to the acceptable difference threshold between old and new policies during gradient descent updates. A large epsilon can increase training speed but reduce stability.
  Lambda (default = 0.95) is a regularization parameter that affects how much the agent relies on the current value estimate when computing the updated value estimate. Low lambda makes the agent more dependent on the current value estimate, while high lambda makes the agent more dependent on the actual reward value obtained from the environment. A suitable lambda can make the training process more stable by balancing the two.
  Num Epoch (default = 3) refers to the number of passes through the experience buffer when performing gradient descent. Large batch size allows for large Num Epoch. Decreasing Num Epoch can make the training process more stable, but reduce the training speed.
  Time horizon (default = 64) corresponds to the experience steps collected before each agent is added to the experience buffer. When the step limit is reached, the total reward value obtained by the current agent will be predicted. Small time horizon means that the agent frequently calculates the current year's reward value and adjusts the strategy accordingly, which is more suitable for scenarios with frequent rewards. Large time horizon should be set to ensure that all important actions of the agent in the episode are collected.

#### 3.4.2. Network Settings

  Hidden units (default = 128) are the number of units in the hidden layer of the neural network. Its size depends on the complexity of the problem, and should be set larger when there is a complex relationship between agent actions and observed variables.
  Num layers (default = 2) are the number of hidden layers in the neural network, which corresponds to how many hidden layers exist after observing the input or after the encoding of visual observations. Fewer layers can achieve better training efficiency, but for complex training environments, more layers are necessary.

#### 3.4.3. Reward Signals

  Gamma (default = 0.99) corresponds to the discount coefficient of future rewards, which affects how much the agent pays attention to possible future rewards. A larger gamma will make the agent more “visionary.” Gamma is between 0 and 1.
  Strength (default = 1.0) is the factor multiplied by the reward value given by the environment, usually set by the default value.

### 3.5. Experiment and Analysis

In experiments, we let Model 0 fight against Model 3 directly by using single-phase training approach and multi-phase training approach, respectively. The idea of using the multiphase training method here is firstly let Model 0 fight against Model 2 to 50 M (we call this step the “turning step”), and then let it fight against Model 3. It is worth mentioning that the reward of Model 0 rises quickly before 50 M steps during training, and it starts to converge at around 47 M steps, and thus we choose 50 M as the turning step.

**Figure 5:**
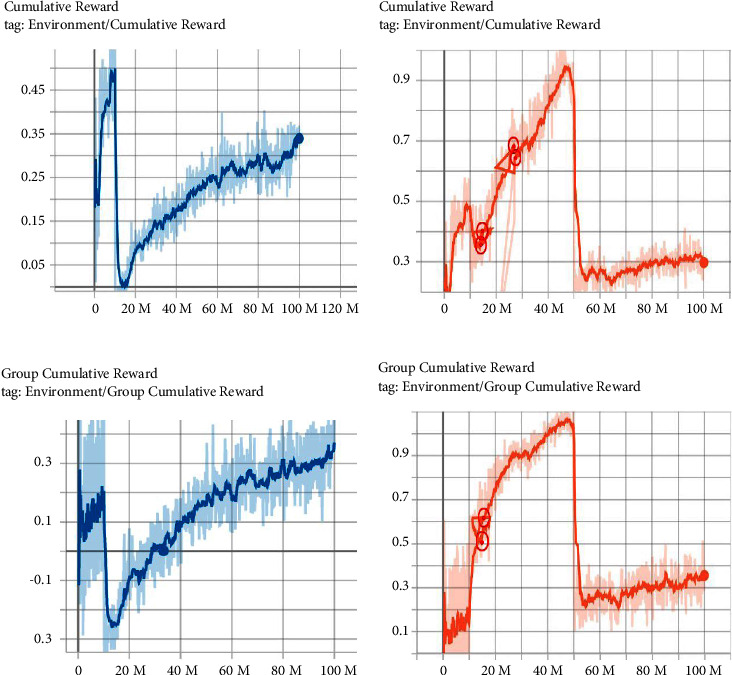
Individual and group reward. (a) Individual reward of single-phase training. (b) Individual reward of multi-phase training. (c) Group reward of single-phase training. (d) Group reward of multi-phase training.

By using single-phase and multiphase training from 10 M to 100 M steps, the individual reward and group reward curves are shown in Figure 5. Due to temporary out-of-memory, the training process may stop accidently. In such cases, the training data would be lost for a few steps. However, tensorboard can only draw continuous simulation curves, and it will automatically fill up some invalid data to make the curves continuous for the case of data missing, which explains why the lines in Figures 5(b) and 5(d) seemingly go back at some points. We have marked in Figures 5(b) and 5(d) the true values of the training data by red circles. In addition, multiagent reinforcement learning is different from single-agent reinforcement learning. In the MA-POCA algorithm, by the attention mechanism, the observations and rewards of all team members will be used as input, and the models will be updated together. Since all three individuals share the same model, their individual rewards are the same.

By comparing Figures 5(a) and 5(b), we find that when steps are between 60 M and 80 M, the multiphase and single-phase individual rewards are similar, but the difference between the winning percentage of them is large. However, by comparing Figures 5(c) and 5(d), we also notice that the group reward of multiphase is bigger than single-phase training. The underlying reason is that the individual decision-making of the multiphase trained team are more inclined to sacrifice individual interests to achieve team goals. This fact might also indicate that the individual reward may not as important as group rewards in multi-agent reinforcement learning since the ultimate goal of agents is to achieve group win.

**Figure 6:**
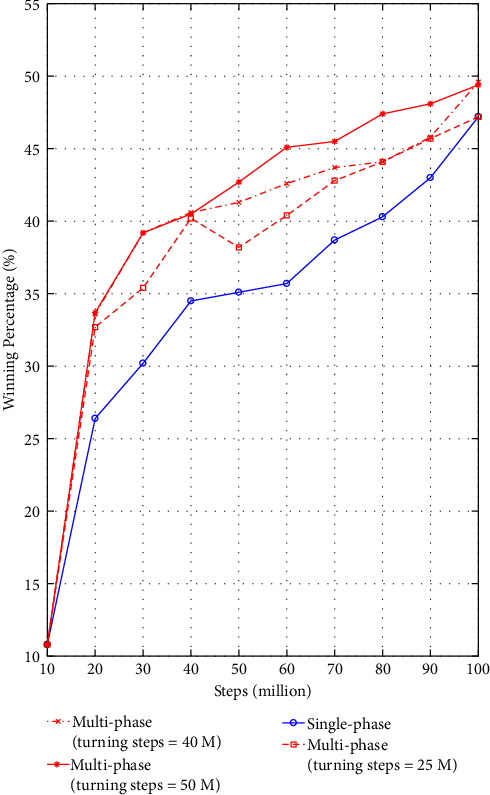
Comparisons between the winning percentages of the single-phase training method and the multi-phase training method.

In Figure 6, it can be observed that the performance of training through multiphase has better performance at each stage. Moreover, we can also find that when at 60 M step, the winning percentage of multiphase training(turning steps = 50 M) reaches 45%, which can be considered to be close to the level of Model 3, but the single-phase training only reaches 45% at 100 M (in fact, the winning percentage at 95 M is only 44.2%). Therefore, multiphase training might reduce the time by 41%–46% compared with single training, which greatly improves the training efficiency. Furthermore, if the trained model is expected to achieve a winning percentage of 47%, multiphase training only needs 70 M steps but single-phase training takes 90 M, which means that single-phase training still needs 28.6% more time.

In addition, we also conduct multiagent training with turning steps of 25 M and 40 M. As expected, they do not perform as well as the case with turning steps of 50 M. As shown by Figure 6, the “25 M” model is much weaker than “50 M” model in the way that it can only break 45% winning percentage at 90 M. By comparison, the “40 M” model is better than “25 M” model on average but still need to be trained to 90 M to reach 45% winning percentage.

From these experiments, it seems that the first-phase training is of great importance as it dramatically inferences the performance of trained model, and a model with poor turning steps may result in bigger impact on the model performance than the impact by inappropriate strategies or parameter configurations. By results of the “25 M” model, the “40 M” model and the “50 M,” it is probably that choosing at the point where the model converges as the turning steps might lead to the best performance.

## 4. Conclusion

In this paper, we build a 3V3 tank confrontation game and the corresponding reinforcement learning environment based on the Unity platform and the ML-Agents reinforcement learning toolkit. The algorithm adopted is MA-POCA which trains tanks to cooperate with teammates and destroy their enemies. We propose a multiphase semistatic reinforcement learning training method, which firstly fixes several strong team without learning and then let the weak team fight against these strong teams to train the weak team. Experiments show that by setting the “turning steps” at the convergence step of the model, the multiphase semistatic method can greatly shorten the training time compared with the traditional single-stage algorithm. Since our study is based on the confrontation of tank groups, which is a typical group confrontation environment, we believe the multiphase semistatic method proposed in this paper might be applicable to other similar group confrontation tasks, such as ball games and MOBA (Multiplayer Online Battle Arena) games. Through further experiments, this training method may prove to be an effective way to address the issues of training efficiency and effectiveness for swarm confrontation tasks.

## Figures and Tables

**Algorithm 1:**
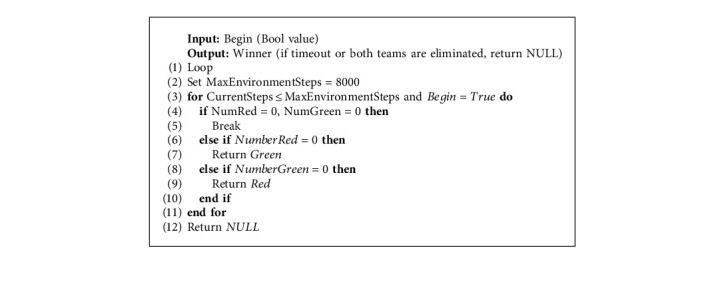
Game rules.

**Table 1:** The attributes of a tank.

Maximum HP	15 points	The angular velocity of the tank rotation	10°/s
The maximum speed of the tank	15 m/s	The field of view distance of the tank	70 m
The angle of view of the tank	360°	Cooling time for firing shells	1.6 s
Maximum damage of shell	10 points	The blast radius of the shell	2 m
Maximum fire speed	15 m/s	Minimum fire speed	10 m/s

**Table 2:** The initial confrontation information of different strong teams vs. the weak team.

Label	Training steps (millions)	Game statistics (win/lose)	Winning percentage
1	106	1373/726	65.4%
2	130	1214/282	81.1%
3	225	1087/132	89.2%

**Table 3:** Individual reward.

Win (alive)	0.6 + 0.6^*∗*^(HP/FullHP) −0.2^*∗*^ResetTimer/MaxEnvironmentSteps
Win (deceased)	0.3
Dead	−0.3
Destroy an enemy	0.15
Collision with tank	−0.05
Collision with wall	−0.5

**Table 4:** Group reward.

Win	0.6 + 0.2^*∗*^(1-2^*∗*^ResetTimer/MaxEnvironmentSteps) +0.4^*∗*^GroupHP/45
Fail	−1.0 + ResetTimer/MaxEnvironmentSteps
Destroy an enemy	0.1
Tie (die at the same time)	−0.2
Timeout	−0.2

**Table 5:** The main parameters of training.

**Hyperparameters**							
Batch size	2048	Buffer size	20480	Learning rate	3.0*e* − 05	Beta	0.01
Epsilon	0.2	Lambda	0.95	Num epoch	3	Time horizon	128

**Network setting**				**Reward signals**			
Hidden units	512	Num layers	3	Gamma	0.99	Strength	1

## Data Availability

The data used to support the findings of this study are available from the corresponding author upon request.

## Abbreviations

ML-Agent: Machine learning-agents
MAS: Multiagent system
RL: Reinforcement learning
DRL: Deep reinforcement learning
MDRL: Multiagent deep reinforcement learning
DQN: Deep Q-network
MA-POCA: Multiagent posthumous credit assignment.
